# ‘It is just a big question mark’: a qualitative interview study of patient experiences of the initial assessment of transient loss of consciousness

**DOI:** 10.1136/bmjopen-2024-098045

**Published:** 2025-03-04

**Authors:** Alistair Wardrope, Lindsay Blank, Melloney Ferrar, Steve Goodacre, Daniel Habershon, Markus Reuber

**Affiliations:** 1 Department of Neurosciences, The University of Sheffield, Sheffield, UK; 2 Department of Neurology, Sheffield Teaching Hospitals NHS Foundation Trust, Sheffield, UK; 3 School of Health and Related Research (ScHARR), University of Sheffield, Sheffield, UK; 4 Syncope and Cardiac Autonomic Service, Sheffield Teaching Hospitals NHS Foundation Trust, Sheffield, UK; 5 Specialised Cancer Services, Sheffield Teaching Hospitals NHS Foundation Trust, Sheffield, UK

**Keywords:** Epilepsy, QUALITATIVE RESEARCH, ACCIDENT & EMERGENCY MEDICINE, Functional Neurological Disorder, Patient Satisfaction

## Abstract

**Objectives:**

Transient loss of consciousness (TLOC) is one of the most common neurological complaints in the Emergency Department (ED), but little is known about the patient perspective. We aimed to explore patient perceptions of diagnostic assessment for TLOC.

**Setting:**

ED, Acute Medical Unit and Syncope and Neurology clinics in a single tertiary teaching hospital in the north of England.

**Participants:**

20 adult patients (60% female, age range 17–90 years) attending or referred with a first presentation of TLOC.

**Primary and secondary outcome measures:**

Exploratory thematic analysis of semistructured qualitative interviews.

**Results:**

We identified three themes within the data: satisfaction with care, unanswered questions and being left in limbo/no man’s land. Participants explored these themes through four topics: communication; the role of investigations; the role of authority and the social context of care.

**Conclusions:**

Communication (including differential diagnosis, significance of investigations and further assessments, and interim safety advice) is emphasised in supporting ongoing self-management, even before a definitive diagnosis is made.

**STRENGTHS AND LIMITATIONS OF THIS STUDY**

- Presents experiences of patients with a first presentation of transient loss of consciousness.
- Reflects experiences of patients with a range of ages and genders.
- Reflexive thematic analysis identifies recurrent themes and foci in patient experience while maintaining contact with issues of greatest relevance to clinicians.
- Exploratory analysis identifies themes of relevance for future research, but limits in-depth discussion of specific issues.
- Lack of concurrent quantitative assessment of patient quality of life prevents mixed-methods analysis that would enrich understanding.

## Introduction

 Transient loss of consciousness (TLOC) is a common Emergency Department (ED) and Acute Medical Unit (AMU) presentation.^1^ Over 90% is due to syncope, epilepsy or functional/dissociative seizures (FDS, also known as ‘psychogenic nonepileptic seizures’ or ‘non-epileptic attack disorder’).^2^ Accurate differentiation can be challenging; at present, 20–30% are initially misdiagnosed, while others receive no working diagnosis at first presentation.^3 4^ This has implications for health and everyday life: patients who could be reassured that they have experienced uncomplicated syncope are told they cannot work or drive pending expert assessment; patients who should be investigated by cardiologists are referred to neurologists and vice versa; and investigations for potentially life-threatening pathologies are delayed. Patients are left in positions of uncertainty.^13 5^,^7^

Professional guidance thus emphasises early specialist involvement; for example, the UK’s National Institute for Health and Care Excellence recommends that all patients with a suspected seizure are reviewed within 2 weeks by a clinician with a special interest in epilepsy for diagnosis, and that all except uncomplicated syncope presentations are assessed by a syncope specialist.^8^ The European Society of Cardiology (ESC) guidelines provide reference for clinicians on assessment of syncope risk and information prior to specialist assessment.^9^ However, this guidance does not address patient experience of TLOC assessment, nor implications for patients of the interval between initial presentation (usually to expert generalists in primary or emergency care) and TLOC specialist (typically cardiology or neurology) assessment.

There is a lack of research aiming to understand patients’ experiences of first TLoC assessment. Previous explorations of patient experiences of seizure care pathways in primary and emergency care more generally have emphasised the importance of service responsiveness, efficiency and continuity, while information and support and consistent communication emerged as particular patient priorities.^10^ People with syncope have been found to prioritise clarity surrounding their diagnosis, report insufficient communication, but prominently report needing to be seen, heard and cared about by their assessing team.^11^

However, the emphasis on specialist diagnoses may have had the unintended consequence of causing generalist clinicians to become deskilled or to lack confidence in assessing such presentations.^12^,^14^ In one study of patients referred to a tertiary TLOC unit, 51% had no provisional diagnosis at the time of referral; of those with a provisional diagnosis, 80% nonetheless considered their episodes ‘unexplained’.^15^

### Research question

To improve clinical care pathways, it is important to understand patient experiences and needs of the assessment process.^16^ Given the lack of previous research on this topic, we therefore conducted an explorative study of salient issues within the initial assessment for patients with a first presentation of TLOC. We sought to explore:

- Overall impressions of the initial assessment.
- Primary patient needs from the initial assessment.
- Holistic life impact of a first presentation of TLOC and its assessment.

## Methods

### Methodology and design

This study comprises a concurrent nested qualitative study within a quantitative project calibrating and validating a differential diagnostic tool for TLOC.^17^ We recruited interview participants from the larger pool of participants recruited to the quantitative study.

### Setting and participants

#### Setting

We conducted this study within a single large teaching hospital trust in the UK, with an adult major trauma centre ED, and tertiary neurology and cardiology services, recruiting from 10 February 2022 to 9 January 2023.

#### Participants

One team member (DH) screened all patients presenting to the ED or AMU with TLOC, and all new referrals to first seizure and syncope clinics, within the window of 10 February 2022 to 9 January 2023. Eligibility for the quantitative study (which involved completion of an online questionnaire by the patient and, if available, a witness) was assessed according to the following criteria.

##### Inclusion criteria

- Patients first presenting with TLOC, with no previous specialist assessment of TLOC.
- Referred to secondary care for diagnostic evaluation; OR given firm diagnosis of syncope in accordance with ESC guidelines for syncope presentations not requiring further investigation.^9^
- Adults over the age of 16 years.
- Able to complete the English-language questionnaire (used in quantitative study) independently.
- Able to give informed consent to research participation.

##### Exclusion criteria

- Previous specialist (neurological or cardiological) assessment of TLOC.
- Secondary cause of TLOC identified.

DH approached eligible patients either in-person (if still in hospital or at clinic) or via retrospective letter, providing them with information on the study and inviting them to participate. Participants could give initial consent at invitation, with consent confirmed prior to completion of the quantitative questionnaire.

When seeking consent for participation in the quantitative study, we separately sought consent for contact to be approached for qualitative interviews. From those who gave consent to contact, one team member (DH) recruited a convenience sample of participants, aiming for a diverse mix of age and genders, approaching them by telephone.

On the basis of empirical studies showing that 24 participants reliably achieve saturation, the narrow specification of subject matter and the study team’s expertise in the subject matter, we expected to reach data saturation within a provisional target of 30 participants.^18^

We determined final diagnoses by two expert reviewers of all clinical data at the end of follow-up, at least 6 months after initial presentation. As we sought to perform interviews early to capture initial experiences most effectively, diagnoses had not been confirmed at the time of approach, and so we could not aim for representativeness across diagnoses.

### Interviews

Two researchers—AW (a speciality registrar (resident) in neurology, with extensive prior clinical experience working as a core trainee (junior resident) in ED and cardiology) and DH (a non-medical healthcare researcher with a background in qualitative and quantitative health research)—conducted semistructured interviews following a predefined interview schedule (online supplemental appendix 1). We conducted all interviews remotely either via video call (Microsoft Teams) or telephone call due to the COVID-19 pandemic. We recorded interviews (either via recording mic or with Teams in-built recording) with explicit patient consent for recording and transcription. Recordings were transcribed by a professional, academic non-clinical transcription service. Interviewers noted initial reflections in contemporaneous logs to support reflexive engagement with later analysis.

### Analysis

We used all interview transcripts as data for thematic analysis^19^ following a reflexive approach,^20^ which we adopted in light of our exploratory aims and experiential focus. In this approach, we did not use a prespecified codebook; one researcher (LB; an experienced healthcare qualitative researcher with no clinical background and no prior personal or professional TLOC experience) imported transcribed interviews into NVivo, coded transcripts and developed initial themes. Two researchers (LB and AW) used these initial themes, alongside prior beliefs and knowledge, in iterative data coding for refinement of the themes. We identified recurrent foci of discussion within and across themes as topics. We then discussed themes within the wider research group prior to final development.

We conducted interim analyses to assess for saturation at various recruitment points and ceased qualitative recruitment when saturation was deemed to have been achieved.

### Reporting

We structured reporting according to the Standards for Reporting Qualitative Research (SRQR); we included the SRQR checklist in online supplemental materials.^21^

### Patient and public involvement

We involved patients at all points. We sought feedback on the study protocol from patient organisation partners (Epilepsy Action, FND Hope, Syncope Trust and Reflex Anoxic Seizures), revising it in light of input. We maintained patient and public involvement oversight through recruitment of a Research User Group, who reviewed study resources; responded to study progress; reviewed results and supported dissemination.

## Results

### Participants and demographics

Of 2811 potential participants screened for recruitment, 1181 were eligible. Of these, 186 responded to the invitation to participate, and 133 also consented to approach for interview.

**Figure 1:**
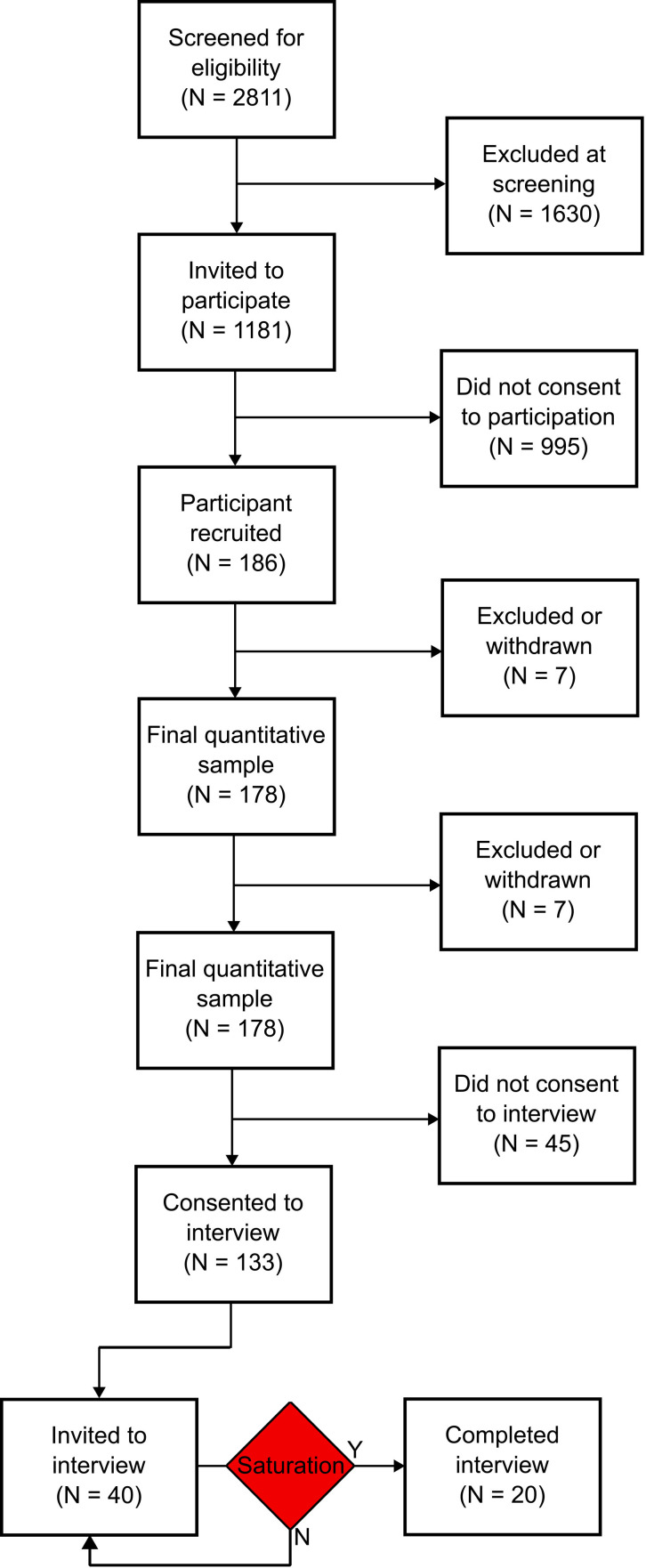
Participant flow diagram.

We approached 40 participants for interview. After 20 interviews, we achieved data saturation. Figure 1 shows the participant flow through the study.

Of interview participants, 14 (70%) received final diagnoses of syncope, and 6 (30%) epilepsy. 12 participants (60%) were female. Median age was 69 years (IQR 39–74.25 years, range 17-90 years). Interviews were held a median of 69 days (IQR 39–117, range 35–283) from initial presentation. 19 patients (95%) presented via the ED, the remainder via Same Day Emergency Care (SDEC; ambulatory general medical assessment unit).^22^ Five patients (25%) were discharged after initial assessment, seven (35%) referred for outpatient specialist assessment (six neurology, one cardiology), the remainder (40%) were admitted or attended SDEC.

### Themes

We developed three core themes (patterns of shared meaning, united by a central concept or idea). Across these themes, we identified 4 cross-cutting topics (foci of discussion),^20^ providing 12 subthemes altogether. Table 1 summarises the themes and topics.

**Table 1:** Summary of themes and topics

Topics	Themes		
	Satisfaction with care	Unanswered questions	Limbo/no man’s land
**Communication**	Perceptions of attention	(Dis)empowering explanations	Communicating plans
**Investigation**	Perceptions of activity	Need for investigations	Awaiting investigations
**Authority**	Joined-up care	Accepting uncertainty	Awaiting experts
**Social**	Systemic pressures	Alternate sources	Social implications

#### Theme 1: satisfaction with care

The majority of respondents were satisfied with the care they received. Where shortcomings were identified, these were attributed to systemic pressures within the National Health Service (NHS). (Dis)satisfaction with care was driven by a need for communication (topic 1); desire for investigation (topic 2); appeals to different authorities (topic 3) and the social context (topic 4) of care; illustrative quotations are given in table 2.

**Table 2:** Illustrative quotations for theme 1

Subtheme	Illustrative quotations
1.1. Perceptions of attention	*I thought when I went to A & E that it would be kind of just, just seen as that. I did, I think, get an ECG scan. I did feel listened, listened to and like notes and that were, were taken*. (TL169) [The GP] *basically wanted to talk about […] sleep apnoea and they just kind of took me away and asked a few questions […] they set up like a double appointment and then I was literally in there thirty seconds*. (TL176)
1.2. Perceptions of activity	*During [my time in the ED] there was, there were tests and things going on all the time, you know, I wasn’t just hanging around, it was […] really impressive*. (TL099)
1.3. Joined-up care	*I’m quite close to my GP cos he’s been my GP since I was a child, so I keep him updated on anything […]. So he’s trying to push me to get into the major head trauma department, [and] neurology assessment as a step forward*. (TL174) *(A)month later(I)ended up at the point we could have been at […] if the two teams had […] agreed that that was a sensible course of action … it was just frustrating that we felt we needed an MRI, […] the medical team felt there needed to be an MRI and the shoulder team said “Absolutely no way is this happening.* (TL184)
1.4. Systemic pressures	*That initial going to A & E, that was difficult […] I’m obviously aware why the wait times […] are so long at the moment, but […] it was tough having to stay in there so long when I […] had no idea really what was going on.* (*TL176*) *I’ve got sort of private medical insurance through work […] and […] I’m starting to wonder if I should explore that and just see if there’s any value in following that up […] it would be nice to […], if I’m having tests, have them sooner rather than later*. (TL188)

##### 1.1. Perceptions of attention

Respondents felt satisfied with care when attended to by staff. The act of note-taking in response to a patient’s story showed concerns were being heard (TL169). Conversely, dissatisfaction arose when respondents did not feel their concerns were being addressed adequately. Multiple respondents reported this occurring when clinician and patient priorities diverged (TL095; TL176).

##### 1.2. Perceptions of activity

Alongside attention, clinical activity—in particular, performing investigations—was considered a metric of good care. Multiple respondents couched their satisfaction in terms of clinicians having “checked everything possible” (TL178). Conversely, some respondents (TL174; TL184) expressed dissatisfaction as a result of investigations they felt were indicated (in both cases advanced imaging) not being performed.

##### 1.3. Joined-up care

Such clinical activity needed to be coordinated between services with different perceived levels of expertise. Primary care played diverging roles: for one respondent (TL174), their general practitioner (GP) served as champion, securing them access to specialist care; for others, the GP was distant (TL176), or even implicated in the cause of their TLOC through lack of coordinated care (TL101). One respondent felt a disagreement between two inpatient care teams delayed their ongoing management (TL184).

##### 1.4. Systemic pressures

Overwhelmingly respondents attributed shortcomings in their care to systemic pressures rather than individual clinical failings. Long waiting times in the ED, for specialist clinic appointments and outpatient investigations, were attributed to staff and resource shortages. Two respondents (TL184, TL188) supplemented their NHS care with private provision.

### Theme 2: unanswered questions

Respondents mostly felt that they did not have a clear understanding of what had happened and did not receive adequate information regarding the cause of their TLOC. Communicating intelligible explanations (topic 1) that supported self-management could address this, as could authoritative reassurance (topic 3); in the absence of this, there was an expectation that investigations (topic 2) could provide the sought-for answers. Without these, some turned to alternative sources of information in their broader information environment (topic 4), but expressed ambivalence about what could be found there. Illustrative quotations are given in table 3.

**Table 3:** Illustrative quotations for theme 2

Subtheme	Illustrative quotations
2.1. (Dis)empowering explanations	*[It] almost clouds the issue that […] my blood pressure dropped […] whether that was as a result of the pain and that was my body’s way of, of managing it, I don’t know, but in my mind it’s, it’s the pain that’s caused the blackouts.* (TL184) *I think they were just basically assessing for concussion but they didn’t really like explain anything about why I might have fainted or like follow-up or anything like that […] I don’t know what caused it […] I’ve tried all sorts to stop it from happening but nothing stops it*. (TL095) *I just wonder if I can do anything else to avoid the situation cos I don’t want to end up in A&E again*. (TL180)
2.2. Need for investigations	*But I’ve had lots of scans and things and tests and you name it and I’ve no idea what’s going off; I wish I do*. (TL146) *[A]t the [district general hospital), he, he just said that “Oh well” he said “we can’t find anything so” he, he said, you know “that’s it.” I was sort of; what do you call it? Dismissed or…* (TL109)
2.3. Accepting uncertainty	*[N]o-one seems to know why […] it happened. But I guess if, if they come to the conclusion there’s not a problem then it, it was purely an isolated occasion and hopefully it won’t happen again*. (TL150) *I think that they provided me with enough clarity on what they thought had happened. I mean they couldn’t obviously pinpoint what had happened, but they just put it down to an unfortunate incident […] I thought OK, yeah, these things can happen, they do happen and I’m just gonna move on, I’m not gonna dwell on it and, yeah, just carry on as I am*. (TL152)
2.4. Alternate sources	*Q: And when they let you out of hospital did they tell you what they thought had caused your blackout? A: Not really, they […] used some words I didn’t quite understand and I can’t quite remember, but […] personally I put a lot of it down to the fact that it was the week where we had all the very hot weather and I’ve never been sort of good with hot weather, and I think it was an accumulation of that*. (TL106) *I’ve […] done quite a lot of Googling, but, […] not come up with anything […] not anymore explanations or how I can avoid it.* (TL180)

#### 2.1. (Dis)empowering explanations

Respondents who felt that their condition had been explained to them in ways they could assimilate into their illness understanding were empowered to self-manage their condition and return to their lives. Those who lacked such explanation felt it more difficult to manage the distress of the original incident (TL109) or mitigate against future occurrences (TL180).

One respondent (TL181) presented an initial explanation of their symptoms (as being a transient ischaemic attack (TIA)) dissonant to the assessing clinician’s (who felt she had experienced a syncopal episode). In contrast to those who felt dissatisfied due to a lack of investigation (1.2), the effort taken by her clinicians to explain how her symptoms did not fit those of a TIA, but could be fully explained by syncope, helped her acceptance of this diagnosis and resolution of the tension.

#### 2.2. Need for investigations

The emphasis on medical investigations as providing an observer-independent—and thus definitive—explanation recurred. Many felt they would not have required answers until they either underwent further investigations or received results of those already performed.

Many felt clinicians—particularly generalists—placed a lot of weight on investigation results. Awaiting investigation was frequently cited as a reason for diagnostic delay (see 3.2 below). Within this investigative paradigm, negative tests could become a barrier to legitimate diagnosis, with patients being “dismissed” (TL109) when investigations failed to yield a positive diagnosis.

#### 2.3. Accepting uncertainty

While most participants had unanswered questions regarding their TLOC, some felt able to move beyond it even in the face of uncertainty. Key to accepting this uncertainty was reassurance from someone they deemed sufficiently authoritative that sinister causes had been excluded and they could be “*reassured […] that it was just a one-off incident*” (TL152). Sufficient exploration of other causes, integration of negative or reassuring test results into the narrative, and concordance with the respondent’s own recovery and lack of ongoing symptoms, were cited factors in accepting such reassurances.

#### 2.4. Alternate sources

Facing unanswered questions, several turned to other sources of information. Some felt they had sufficient understanding that they could explain their TLOC without the clinician’s endorsement, particularly given they “*didn’t understand*” and “*can’t quite remember*” the clinician’s account (TL106). Otherwise, the most commonly cited alternative was information gained through internet search engines (TL169, TL180, TL188). Generally participants expressed ambivalence about information thus obtained, being concerned about its reliability and potential adverse effects (TL169), or finding that it failed to enhance understanding (TL180). Those who were able to accept uncertainty regarding their TLOC felt less need to pursue alternative information sources (TL188).

### Theme 3: limbo/no man’s land

To many respondents, the experience of TLOC was of an abrupt change in their experience of and relation to the world; in one respondent’s words, “*twelve hours before I’d been absolutely fine and now everything had changed overnight*” (TL176). Assessments often failed to achieve resolution, leaving respondents in an interstitium variously described as “*limbo*” (TL146) or “*no man’s land*” (TL157). This stasis was exacerbated in cases where respondents had no clear understanding of what they were being referred for (topic 1). This waiting phase was attributed to the need both for investigations (topic 2), and for specialist input (topic 3); some described multiple primary care contacts, the upshot of which would be simply being told to “*just wait for Neurology”* (TL122); this waiting time could be prolonged (a median wait of 48 days in the UK and Ireland).^23^

In this condition, respondents struggled to negotiate social implications (topic 4). Some reported possible diagnoses that restricted their activities (notably driving), without the social licence granted by a definite diagnosis. Those equipped with empowering explanations (2.1) or the ability to accept and manage uncertainties (2.3) experienced less disruption. Illustrative quotations are given in table 4.

**Table 4:** Illustrative quotations for theme 3

Subtheme	Illustrative quotations
3.1. Communicating plans	*[T]hey gave me […] a leaflet about seizures and said […] it’s a working diagnosis, it’s […] not confirmed yet … … so that’s kinda where I’m at, I’m a bit in no-man’s land at the moment, I’m not entirely sure what’s going on*. (TL157) *[T]his kinda period of, of not knowing what it, what it is and not knowing when you’re kind of gonna be seen it […] makes it a little […] bit difficult cos obviously I don’t want to have to keep going to my GP or the A&E […] every time I experience these episodes*. (TL169)
3.2. Awaiting investigations	*I had to ring my GP to find out is there a plan […] and they were like “Well we don’t know at the moment; they’re doing their investigations.* (TL157)
3.3. Awaiting experts	*I went to the GP, I think it was earlier this year, around April/May time, and they recommended that if I had another kind of episode to, to go to A & E. I think I went to A & E a few months back and I think they did an ECG and I was just advised to go home and wait for an appointment with the neurologist*. (TL169) *They just kept an eye on me again and did some blood tests and everything come back fine. So they said “Just wait for neurology, try and get a quicker appointment.”* (TL122)
3.4. Social implications	*[I]t’s difficult to […] speak about it with friends because it […] is just a big question mark. But it is really starting to […] affect my life*. (TL169) *[M]y experience with the seizure nurse, I feel like that has definitely exceeded my expectations.[…] she was really knowledgeable, she really, you know, explained everything […] she expanded on a lot of the things I could […] and couldn’t do and […] I had to be careful with. […] I do feel like if I’ve got any questions that she’s […] my first point of call to go to*. (TL176) *[I] t’s been quite […] life changing […] I’m gonna have to go part-time at work […] I think a follow-up a week later, even just a phone call, would have been really helpful*. (TL157)

#### 3.1. Communicating plans

Several respondents felt clinical activity was happening around them, but without involving them, leaving them feeling unable to proceed despite believing the clinical teams may be closer to a diagnosis or management plan. Many reported being informed of the need for follow-up, but fewer were clear why they were being referred (TL188). Interim communication of information (working diagnoses, information leaflets) went some way to address this, but incompletely (TL157). Respondents were unsure how to self-manage in the interim without further information being shared (TL169).

#### 3.2. Awaiting investigations

Some respondents reported difficulties in making progress while awaiting outstanding investigations. Results of the investigations were a barrier to progress both for patients (TL099) and clinicians (TL157).

#### 3.3. Awaiting experts

Respondents felt stuck waiting for specialists. The outcome of initial assessments was often not a provisional explanation or interim management plan but a recommendation to await specialist assessment. Some felt passed between primary and emergency care (TL169), while long secondary care waits left them with no clear end in sight (TL122; theme 1.4). Patients were unwilling to re-present in the interim as they expected they would just be advised to await a specialist again (TL169, TL173).

Some respondents had already undergone specialist assessment by the time of the interview; while contact with these services helped to resolve the feeling of stasis for some (eg, TL176), others reported remaining in a similar situation (TL146), even with a provisional diagnosis.

#### 3.4. Social implications

The social implications of TLOC were particularly difficult to manage in this limbo period. For some, these related to specific practical limitations, for example, driving restrictions (TL176) or work modifications (TL157); for others, ongoing symptoms affected their career path (TL174).

Others encountered the social implications of lacking a diagnosis, their TLOC being a “big question mark” (TL169). Clear diagnoses would allow people to determine what may or may not need to change in their lives, whereas being in limbo imposed restrictions without associated social licence for deviation from their previous role (TL146).

For respondents who had undergone specialist assessment, available services proved valuable in negotiating these social challenges; one respondent (TL176) singled out the role of the epilepsy specialist nurse in navigating the psychosocial as well as biomedical ramifications of their condition.

## Discussion

This study explores patient experiences of the initial assessment of TLOC—of the care delivered, and its impacts on their subsequent life. For many, this was significant; “*twelve hours before I’d been absolutely fine and now everything had changed overnight*” (TL176). We interpret this as TLOC presenting a “biographical disruption”^24^ that demanded major re-evaluation of personal narratives and social circumstances. This disruption could be ameliorated if respondents were reassured—by appropriate clinical authorities, and ideally with evidential support from investigations—that the event was an “unfortunate one-off incident” (TL152; 2.3). Prolonged delays to specialist assessment drew out this disruption and its psychosocial consequences.

### Satisfaction with care

Respondents were largely satisfied with the care they received at their initial TLOC assessment. Drivers of (dis)satisfaction included feeling paid attention to and feeling heard; having investigations performed; being kept informed and (lack of) prolonged waits. After the initial assessment—but before the specialist review—informational needs drove satisfaction, with a lack leaving respondents with unanswered questions (theme 2) or feeling left in limbo (theme 3).

Respondents used the extent of investigation as a barometer of good care (1.2); many of their unanswered questions related to investigation results (2.2); and clinicians and respondents alike found it difficult to proceed until investigation results returned (3.2). This is despite evidence that, in the assessment of TLOC, investigations are of limited utility—typically normal or misleading.^25 26^ Patient and witness histories of the TLOC remain the cornerstone of diagnosis,^27^ but respondents felt less comfortable with a focus on such accounts over test results (eg, considering the history “*my interpretation”,* whereas investigations are *“an objective method, […] of seeing what I, how I am”* (TL099)). Two respondents were specifically dissatisfied due to not receiving what they perceived to be necessary investigations—in both cases, advanced imaging. However, such imaging is rarely of much benefit in supporting the differential diagnosis of TLOC; in one study of older adults presenting with TLOC, CT or MRI was performed in 63% of presentations, but only in 2% of those was any abnormality found (overwhelmingly when such abnormalities were expected based on other symptoms or examination findings).^28^

However, the authority of investigations was not absolute; one respondent (TL181), who initially felt she needed investigation for a TIA, was instead reassured by her assessing clinician’s explanation of how her presentation fit far better with syncope. Effective communication with empowering explanations of the cause of TLOC that supported self-management (2.1), especially when delivered by individuals perceived as having relevant authority (2.3, 3.4), could support patients in navigating the biographical disruption.

Our findings are consonant with Clouser *et al*’s research into the experiences of patients with syncope (the majority of whom had chronic/recurrent presentations). Their respondents also reported that clinicians demonstrated their care and attention both by clear communication and aggressive investigation, and that they sought clear explanations.^11^ Similarly, people with suspected seizures describe largely consonant needs, emphasising the value of time being available for care and the issues of waiting, the need for care continuity and the cross-cutting importance of communication.^10^ Our findings enhance their conclusions by demonstrating that similar concerns are relevant across TLOC-causing presentations (rather than just syncope or seizures) at first presentation (rather than in chronic/recurrent populations), and that the topics of communication and testing recur across broader themes including the holistic, social impact of TLOC on patients’ lives.

### Impact of TLOC on patients’ lives

Our respondents demonstrate the holistic impact of TLOC beyond the paroxysmal event itself. By the time of the interview, most were asymptomatic (some experienced symptoms related to comorbidities or from complications of their TLOC, eg, injuries sustained in collapse). Nonetheless, TLOC provides a striking discontinuity in many respondents’ narratives, an abrupt disruption that, unlike most chronic illnesses, does not ‘creep up’ on the patient but throws them into a ‘critical situation’.^24^ Even respondents who felt less disabled by their TLOC refracted their future plans through the lens of biological considerations appropriate to the explanations afforded for their TLOC (2.1). This transformation undermined confidence in their independence, leaving them feeling adrift with significant social implications (theme 3). These findings are consonant with the quantitative evidence that people with syncope—even when not recurring—score lower on quantitative measures of health-related quality of life (HRQoL) than reference populations, and show a high degree of disability (with a mean functional impairment in 33% of listed activities).^29^ Furthermore, this impact—while ameliorated—persists at 1 year from initial presentation.^30^

We also found that people who experience TLOC need tools to navigate this disruption. National guidance mandates that people who experience TLOC merit referral for specialist assessment, but does little to address their interim needs—for example, explanations to support empowered self-management (2.1) or help in navigating the social implications of possible diagnoses (3.4). Diagnoses like epilepsy have a range of associated psychological and social implications (eg, employment and driving).^5 31^ Our respondents frequently described situations where they were informed they might have epilepsy—and thus faced the associated psychosocial implications—but had no firm diagnosis that would allow them to access the available resources to help them navigate these life changes. In some cases, normal investigations blocked the process of the patient’s experience becoming a medical concern (with associated social value), leaving the patient “*dismissed*” (TL109; 2.2).

### Implications for practice

Our study provides novel insights into the care needs of this high-incidence patient group. Our data are largely consonant with Graham *et al*’s typology of ED patient needs, emphasising their communication needs (both in feeling heard and being given information); their emotional needs (tools for managing uncertainty and empowering self-management); competent care needs and waiting needs (which our respondents were very careful to attribute to systemic and political problems, rather than individual staff shortcomings).^16^ The social and psychological implications of the biographical disruption created by the TLOC event extend beyond the individual occurrence, however, and our respondents demonstrated how empowering explanations (2.1) and tools to manage uncertainty (2.3) provided at initial assessment could be valuable in meeting their needs in the immediate aftermath. Provision of information resources to allow exploration of their understanding of TLOC, its causes and the assessment process may help meet these needs in the interstitium between initial presentation and specialist assessment. Reducing barriers to specialist assessment, by improving timely access to appropriate specialist services, is clearly also required; our respondents showed the significant benefit of talking with professionals with relevant expertise (eg, seizure nurses).

### Limitations and reflexivity

It is important to note some limitations to our study. Most prominently, as an exploratory study the themes developed and topics identified are relatively high level. We found themes saturated at 20 participants, but a richer interview protocol interrogating each of our themes in further detail could enhance our findings. This would particularly be complemented by quantitative measures of some of our respondents’ main concerns, such as holistic impact (eg, through measuring HRQoL), or level of functional impairment (eg, by using the ICF-2001 framework).^32^ Previous quantitative research has demonstrated the impact of syncope on HRQoL,^29 30^ but mixed-methods exploration could enhance this approach and ours.

While we aimed for a diverse mix of gender, age and diagnosis in our sample, the nature of the study and the interviews meant that our interview sample is not representative of the entire population experiencing TLOC. Most notably, given that we aimed to conduct interviews sufficiently soon that participants were still able to recollect their initial presentation, we did not have final diagnoses available when recruiting participants for interview. As such, we did not recruit any patients with a final diagnosis of FDS to interview. This was the least frequent diagnosis in our study, and the relative proportions of syncope and epilepsy patients in the interview sample reflect their frequencies in the wider study population. From other qualitative studies, we know that people with FDS report similar experiences—notably that of being left in limbo—to those reported here.^33^ Additionally, those with clinically certain uncomplicated syncopal presentations were under-represented in our sample, which may have made themes of uncertainty and delay more prominent.

Beyond this, we were limited to people with sufficient English-language comprehension to participate in the quantitative study; this not only means that some communities’ voices are not captured in this study, but also that certain patient groups in whom TLOC is particularly prevalent (eg, those with cognitive impairment or intellectual disability) are not captured here. Further work should seek to amplify these voices.

The quantitative study (of which the sample for this study is a subgroup) also had a low overall recruitment rate, with just 15.7% of those deemed eligible at screening consenting to participate. If there were systematic differences in recruitment by diagnosis (or, eg, patient demographics), this would bias the external validity of our results.

We acknowledge that the prior expectations and background of the research team will have shaped the interpretation of our results; we view this as inevitable, but take measures to allow our results to temper these biases. The semistructured nature of the interviews allowed participants to direct dialogue down paths not proposed by the interviewer. One interviewer (DH) and the team member who performed initial development of themes (LB) were not clinicians or people who would otherwise work with people experiencing TLOC, thus were less subject to clinical biases; this was balanced with interview and analytic input from AW, and subsequent revision from the rest of the study team, who brought specific neurology, cardiology and ED expertise to refine analysis in view of grounding knowledge. The iterative process of working back and forth between themes and data allows for the ‘surplus of meaning’^34^ within the data when constrained to any particular coding to reshape interpretation; the themes described above represent an equilibrium reached through this hermeneutical circle.^35^

## Conclusion

Communication (including differential diagnosis, significance of investigations and further assessments, and interim safety advice) is key to supporting ongoing self-management for people who experience TLOC, even before a definitive diagnosis is made. Much recent research—including the quantitative arm of this present study—has been focused on developing tools to maximise information available to the clinician in the differential diagnosis of TLOC; future work should explore whether such tools could also be used to support patients in understanding their presentation, assessment and interim self-management while awaiting a definitive diagnosis and management plan.

Beyond this, even after extensive investigation and specialist review, TLOC remains a challenging presentation and many patients will not receive a clear diagnosis or definitive management plan. Such uncertainty is pervasive throughout clinical practice, and our respondents highlight how important it is for patients that clinicians acknowledge and manage that uncertainty.^36^ Our data show how the promise of further tests or assessments can be used as a delaying strategy, avoiding the need for clinicians to acknowledge the limitations of medical technology and expertise to parse human experience. This process often left people in ‘limbo’, unequipped to manage the uncertainty with which they were left. From even the first point of contact with health services, clinicians working with people experiencing TLOC need to provide them with the resources to understand and manage this uncertainty.

## supplementary material

10.1136/bmjopen-2024-098045 online supplemental file 1

10.1136/bmjopen-2024-098045 online supplemental file 2

## Data Availability

Data are available upon reasonable request.
