# Supplementary material for: ‘It is just a big question mark’: a qualitative interview study of patient experiences of the initial assessment of transient loss of consciousness
Source: BMJ Open. 2025 Mar 4;15(3):e098045. doi: 10.1136/bmjopen-2024-098045 (PMC11881173; doi:10.1136/bmjopen-2024-098045)
Supplement: online supplemental file 1 [file bmjopen-15-3-s001.doc]

**Clinical decision aid for transient loss of consciousness – interview schedule**

Introduce myself and explain that the purpose of the interview is primarily to talk about their views about their presentation, their clinical trajectory, and experience of using the iPEP. I will also ask about any additional questions or modifications they would think suitable.

Complete consent form, answer any questions and remind interviewee they may terminate the interview at any stage, or choose not to answer a question if they prefer.

**Interviewee data:**

Firstly I would like to ask a few questions about you and your condition:

1. Check demographic information

2. Brief background about their current condition

Prompts: How long ago / when did your blackouts start? What happens / what are they like / how often do they happen?

**Perspectives about initial TLoC assessment**

1. How did you first present with your blackouts? (GP, Emergency Department, other)

2. Have you received a diagnosis for the cause of your blackouts, or do you have further assessment/follow-up arranged?

3. Do you feel your assessment thus far has been adequate?

4. Do you feel you have unanswered questions about your blackouts?

5. Do you feel confused about what the next steps are in managing your blackouts?

Prompts: What services, where accessed, how long, how often

**Perspectives on using the iPEP**

1. General opening – how did you find using the iPEP?
2. Do you think the questions were appropriate?
3. Do you think the questions captured the most important aspects of your experiences of your blackouts?
4. Did you struggle to understand any of the questions?
5. Did you struggle to answer any of the questions?
6. How did you complete the iPEP? Did you find it easy to complete?

**Perspectives on improving the iPEP**

1. Do you think using a tool like the iPEP would have improved your experience of your initial assessment with the GP/in ED?
2. Do you think your experience of the iPEP could be improved? How?
3. Would you change the questions of the iPEP?
4. Would you change the interface?

Prompts: Barriers to use; barriers to understanding; overlooked aspects; understanding intended use of iPEP

Thank you for your time today, is there anything else you think might be helpful for me to know / is there anything you want to ask? Thank you…
