# Supplementary material for: ‘It is just a big question mark’: a qualitative interview study of patient experiences of the initial assessment of transient loss of consciousness
Source: BMJ Open. 2025 Mar 4;15(3):e098045. doi: 10.1136/bmjopen-2024-098045 (PMC11881173; doi:10.1136/bmjopen-2024-098045)
Supplement: Supplementary file 3 [file bmjopen-2024-098045.reviewer_comments.pdf]

PEER REVIEW HISTORY

BMJ Open publishes all reviews undertaken for accepted manuscripts. Reviewers are asked to complete a checklist review form and are provided with free text boxes to elaborate on their assessment. These free text comments are reproduced below.

This paper was submitted to another journal from BMJ but declined for publication following peer review. The authors addressed the reviewers' comments and submitted the revised paper to BMJ Open. The paper was subsequently accepted for publication at BMJ Open.

ARTICLE DETAILS

Title (Provisional)

"It is just a big question mark": A qualitative interview study of patient experiences of the initial assessment of transient loss of consciousness

Authors

Wardrope, Alistair; Blank, Lindsay; Ferrar, Melloney; Goodacre, Steve; Habershon, Daniel; Reuber, Markus

---

VERSION 1 - REVIEW

**Reviewer**	**1**
**Name**	**Reviewer 1**
**Affiliation**	
**Date**	**10-Jun-2024**
**COI**	**No competing interest**

Comments not published pending reviewer's consent.

**Reviewer**	**2**
**Name**	**Reviewer 2**
**Affiliation**	
**Date**	**10-Jun-2024**
**COI**	**No competing interest**

Comments not published pending reviewer's consent.

VERSION 2 - REVIEW

**Reviewer**	**1**
**Name**	**Sosa, Ivan**
**Affiliation**	**University of Rijeka Medical School, Department of Forensic Medicine and Criminalistics**
**Date**	**25-Dec-2024**
**COI**	**None**

Please adapt your abstract to accurately represent the article. It should be a single paragraph of no more than 200 words. For research articles, the abstract should provide a relevant overview of the work. We strongly encourage authors to follow a structured abstract format, but without headings. This format should include: (1) Background: Contextualize the question being addressed and highlight the study's purpose; (2) Methods: Briefly summarize the main methods or treatments used; (3) Results: Outline the article's key findings; (4) Conclusions: Present the main conclusions or interpretations. The abstract must objectively represent the article and should not include results that are not presented and substantiated in the main text, nor should it exaggerate the main conclusions.

**Reviewer**	**2**
**Name**	**Pyykko, Ilmari**
**Affiliation**	
**Date**	**06-Jan-2025**
**COI**	**None**

1. Patient Experiences and TLoC Evaluation

Analyzing patient experiences during the initial assessment of Transient Loss of Consciousness (TLoC) is crucial for improving diagnostic accuracy and patient satisfaction. However, publishing analytical papers in this domain is challenging as they traditionally require methodologies based on open-ended questions and classifications aligned with widely accepted models or frameworks. The paper has a rather narrow scope for qualitative analysis but it is valuable to improve the focus of EA activity. The paper is in the present form too long and should be shortened. I recommend that the paper will be published in the journal.

Reviewer Comments and Recommendations:

General Comments

1. **Patient Experiences and TLoC Evaluation**
   Analyzing patient experiences during the initial assessment of **Transient Loss of Consciousness (TLoC)** is crucial for improving diagnostic accuracy and patient satisfaction. However, publishing analytical papers in this domain is challenging as they traditionally require methodologies based on open-ended questions and classifications aligned with widely accepted models or frameworks. The paper has a rather narrow scope for qualitative analysis but it is valuable to improve the focus of EA activity. The paper is in the present form too long and should be shortened. I recommend that the paper will be published in the journal.

This study importantly incorporates patient experiences while adhering to **National Institute for Health and Care Excellence (NICE)** guidelines, which emphasize a structured initial assessment for individuals presenting with TLoC. This approach is vital, as diagnostic tests and clinical evaluations often fail to conclusively identify the cause of TLoC. NICE guidelines recommend recording a detailed clinical history and conducting physical examinations to guide management but from patient perspective it does not provide guidelines for patient rehabilitation and living within uncertainty.

2. **Length of the Paper**
   - The manuscript is overly long Especially for an international audience the patient narratives should be translated into scientific English to minimize bias from misunderstanding of local dialects.
   - Tables need significant shortening for brevity and focus.
3. **Qualitative Data Analysis**
   - The qualitative analysis identifies multiple themes (14 in total) but appears fragmented and not sufficiently synthesized to enable clear conclusions.
   - The study aimed to explore the holistic life impact of a first presentation of TLoC. While this was discussed in the results, it was inadequately addressed in the methodology. Consider integrating a standardized health-related quality of life measure, such as the **EQOL-5D-5L** questionnaire, for more direct insights. Structured questions may better capture patient attitudes and facilitate tailored counseling.

Methods Section

1. **Characterization of TLoC Consequences**
   - The paper acknowledges activity limitations and participation restrictions but lacks detailed analysis. Utilizing the **ICF-2001 framework**

(International Classification of Functioning, Disability and Health, WHO) could enhance this aspect, particularly in assessing functional impairments such as driving restrictions.

  - Positive aspects of TLoC experiences, which can occur even in severe conditions like cancer or AIDS, were largely overlooked. These should be systematically addressed to provide a balanced perspective.

2. **Quantification Challenges**
   - The differentiation between qualitative and quantitative analyses is unclear. A graphical representation indicating which participant groups underwent qualitative versus quantitative analyses would enhance clarity.
   - While major themes and their positive and negative aspects are defined, data saturation with only 20 participants may not be sufficient for robust characterization.

Discussion Section

1. **Comparative Studies**
   - The statement on page 16 claiming novelty ("This study is the first…") is overstated. Similar research has explored patient experiences, including studies cited in this manuscript (e.g., Clouser et al., 2021; Williamson et al., 2021).
   - These studies highlight the complexity of decision-making in syncope evaluation and the importance of patient-centered approaches. Incorporating these findings would contextualize and strengthen the discussion.
2. **Unsubstantiated Claims**
   - On page 16, the interpretation of TLoC as a "biographical disruption" extending to participation restriction is not supported by the study's data. Reframe this as a hypothesis: "We interpret that…"
   - The discussion on "bodily doubt" and its implications for chronic illness (pages 17–18) lacks supporting data. Consider removing this section or explicitly stating it as speculative.
   - The paragraph on page 18 about symptoms as "commodities" and their social implications is not adequately addressed in the study and should be removed.

Outcome Section

The conclusion that patient experiences during the initial assessment of TLoC are shaped by their expectations for communication, comprehensive evaluation, and empathetic care is well-supported. This finding provides a strong foundation for further research into patient-oriented TLoC evaluation strategies, particularly in emergency assessment units.

Additional Comments

1. **Interview Protocol**
    - The lack of access to Appendix 1 (interview protocol) limits a full review of the study's methodology. Ensure the appendix is included in the final paper.
2. **Improving Patient Narratives**
    - Shorten patient reports and ensure their presentation avoids redundancy. Focus on concise themes to enable clearer conclusions.
    - Incorporate direct measures of health-related quality of life (e.g., EQOL-5D-5L) to strengthen the assessment of TLoC's impact on patients' lives.
3. **Future Directions**
    - Develop tools to better quantify qualitative findings, using frameworks like ICF-2001 to classify functional and participation impacts.
    - Expand participant recruitment to achieve data saturation and enable more sophisticated analyses of themes.

**Reviewer**	**3**
**Name**	**Jansen, Sofie**
**Affiliation**	**Amsterdam University Medical Centres, Geriatric Medicine**
**Date**	**12-Jan-2025**
**COI**	**None**

Thank you for letting me review this article. I think the paper is very well written, and the results emphasize how important robust communication is in the context of syncope, especially when further tests are awaited. Ruling out certain causes does not mean that patients have been told what they do have, and the results from this paper highlight that. As much as I want to contribute to the peer review process by suggesting further improvements, I don't have any, so I recommend acceptance of the paper.

VERSION 2 - AUTHOR RESPONSE

REVIEWER 1		
1.1.	Please adapt your abstract to accurately represent the article. It should be a single paragraph of no more than 200 words. For research articles, the abstract should provide a relevant overview of the work. We strongly encourage authors to follow a structured abstract format, but without headings. This format should include: (1) Background: Contextualize the question being addressed and highlight the study's purpose; (2) Methods: Briefly summarize the main methods or treatments used; (3) Results: Outline the article's key findings; (4) Conclusions: Present the main conclusions or interpretations. The abstract must objectively represent the article and should not include results that are not presented and substantiated in the main text, nor should it exaggerate the main conclusions.	We have restructured the abstract in the recommended format, as a single paragraph removing the headings but maintaining reporting of the background, methods, results, and conclusions.
**REVIEWER 2**		
2.1.	The paper is in the present form too long and should be shortened.	The manuscript has been shortened through reducing the size of the tables and removing parts of the discussion highlighted by the reviewer (2.12, 2.13).
2.2.	Especially for an international audience the patient dialects should be translated into scientific English to minimise bias from understanding of local dialects.	We do not wish to modify the data by rephrasing or translating participants' answers, but where necessary have abbreviated quotations for clarity.
2.3.	Tables need significant shortening for brevity and focus.	We have shortened the tables, removing duplicate quotations that covered similar sub-themes.
2.4.	The qualitative data analysis identifies multiple themes (14 in total)	We are grateful to the reviewer for highlighting the diversity of themes in our

	but appears fragmented and not sufficiently synthesised to enable clear conclusions	literature. We have attempted to integrate by noting there are just three key themes within our analysis; however we have used recurrent topics of discussion to synthesise across themes. To make this clearer we have edited the introduction to each theme in the results, to highlight how the topics apply in each sub-theme, and have edited the results to integrate across the themes. We have also amended the discussion (see below) to synthesise themes more.
2.5.	The holistic life impact of a first presentation of TLoC … was inadequately addressed in the methodology. Consider integrating a standardised health-related quality of life measure. Structured questions may better capture patient attitudes and facilitate tailored counselling.	We are very grateful to the reviewer for this excellent suggestion. Unfortunately, this was conducted only as an initial exploratory study, and we identified this holistic life impact as a significant concern only from this exploratory analysis. We agree that our results suggest future mixed-methods exploring this topic in more detail, combining a more targeted interview with quantitative assessment, would be worth pursuing. We have now identified the lack of quantitative assessment in the limitations, and highlighted this as a suggestion for future research.
2.6.	The paper acknowledges activity limitations and participation restrictions but lacks detailed analysis. Utilising the ICF-2001 framework could enhance this aspect.	As with the response to 2.5. above, we definitely feel our results merit further more detailed research exploring the holistic life impact including functional restrictions arising from a first presentation of TLOC.
2.7.	Positive aspects of TLoC experiences … were largely overlooked. These should be systematically addressed to provide a balanced perspective.	We did not find in our data participants reporting positive aspects of their TLOC experiences, beyond those who felt that they did not feel significant life impacts and felt satisfied with their care. We have sought to address the care factors that enabled self-management, but we do not report participants finding TLoC providing positive experience, as this was not present in our data.
2.8.	The differentiation between qualitative and quantitative analyses is unclear. A graphical representation indicating which participant groups underwent qualitative versus quantitative analyses would enhance clarity.	We have inserted a participant flow diagram to clarify this, and are grateful to the reviewer for the suggestion.

2.9.	Data saturation with only 20 participants may not be sufficient for robust characterisation	From iterative analysis we found that by 20 participants our developed themes did not appear to expand, and thus concluded we had reached saturation for this exploratory study. We agree that robust characterisation of themes would merit deeper exploration with more participants, but that this would better be achieved through follow-up studies with more a more structured interview protocol and associated quantitative interrogation (e.g. as discussed in response to 2.5. above).
2.10.	The statement on page 16 claiming novelty ("This study is the first…") is overstated. Similar research has explored patient experiences, including studies cited in this manuscript (e.g. Clouser et al 2021, Williamson et al 2021) […] Incorporating these findings would contextualise and strengthen the discussion.	We still feel that our study is the first looking at first presentations of all TLOC disorders (Clouser et al look only at syncope, and over half of their sample were people with chronic or recurrent syncope, and Williamson et al studied people who did *not* present), but we acknowledge that the prior phrasing did not fully reflect preceding research and so have amended this. We have also embedded further the discussion of prior research throughout the discussion.
2.11.	The interpretation of TloC as a "biographical disruption" … is not supported by the study's data. Reframe this as a hypothesis: "We interpret that…"	We have reframed this portion of the discussion as a hypothesis, though feel that there is significant support for our contention in our data. Multiple respondents described that their TLoC had been "*life-changing*", or that "*everything changed overnight*"; this combined with the evidence of lasting effects on functioning and quality of life now cited in the discussion give us reason to make this statement. We find this result of value, as the biographical disruption literature has hitherto concentrated on chronic illness or progress problems such as frailty; finding that people who experience a single paroxysmal event can similarly have such lasting consequences is relevant.
2.12.	The discussion on "bodily doubt" and its implications for chronic illness … lacks supporting data. Consider removing this section or explicitly stating it as speculative.	We have largely removed this section, though maintained some points enhancing the finding referenced in 2.11. that concepts used to understand chronic illness experience are also of relevance in understanding TLoC.
2.13.	The paragraph on page 18 about symptoms as 'commodities' and their	This section has now been largely removed, though the salient aspects

	social implications is not adequately addressed in the study and should be removed.	regarding holistic impact of TLOC is maintained.
2.14.	The lack of access to Appendix 1 (interview protocol) limits a full review of the study's methodology. Ensure the appendix is included in the final paper	We apologise for this oversight and now include the interview protocol.
2.15.	Shorten patient reports and ensure their presentation avoids redundancy. Focus on concise themes to enable clearer conclusions	We have amended the interview excerpts to avoid duplication and make tables more concise.
2.16.	Incorporate direct measures of health-related quality of life (e.g. EQOL-5D-5L) to strengthen the assessment of TLoC's impact on patients' lives.	As with point 2.5. above, we agree this would be highly valuable in future research, and now reference this in the discussion and limitations.
2.17.	Future directions: develop tools to better quantify qualitative findings	See 2.16. and 2.5. above.
2.18.	Future directions: Expand participant recruitment to achieve data saturation and enable more sophisticated analysis of themes.	See 2.9. above.
**REVIEWER 3**		
3.1.	No revisions suggested	We are very grateful to the reviewer for acknowledging the value of this work and for their generous comments.

VERSION 3 - REVIEW

Reviewer **2**

Name **Pyykko, Ilmari**

Affiliation

Date **31-Jan-2025**

COI

Final comments of reviewer (after revision) on the study "It is just a big question mark": A qualitative interview study of patient experiences of the initial assessment of transient loss of consciousness"

The outcome of the study demonstrates that the patient experiences during the initial assessment of TLoC are influenced by their expectations for clear communication,

comprehensive evaluation, and empathetic care. Healthcare providers can enhance patient satisfaction and diagnostic accuracy by adopting patient-centered communication strategies and adhering to established clinical guidelines. This conclusion is solid and provides a good substrate for clinical work and further analysis and quantification of patient oriented TLOC-evaluation in EA units.

The qualitative analysis of the qualitative data seems to provide several different aspects that were for investigation purposes focused on 14 major aspects (themes and topics). Although positive and negative aspects have been defined in each major themes the analysis may be too limited to allow sophisticated characterizations of the content and maybe to reach data saturation within a provisional target of 20 participants may not be satisfactory.

One of the main purposes was to study the holistic impact of a first presentation of TLOC and its assessment. In results this question was not fully explored but, in the discussion, a major paragraph focused on these questions (Impact of TLOC on patients' lives). In the next study the authors might include in their questions a direct standardized question as EQOL-5D-5L questionnaire.
